# Surgical Epicardial CRT-D Implantation in a Patient with Complete Obstruction of the Superior Vena Cava

**DOI:** 10.5935/abc.20180070

**Published:** 2018-05

**Authors:** Gustavo Lima da Silva, Nuno Cortez-Dias, João de Sousa, Ângelo Nobre, Fausto J. Pinto

**Affiliations:** Centro Hospitalar Lisboa Norte, Hospital de Santa Maria, Lisboa - Portugal

**Keywords:** Heart Failure, Tachycardia, Ventricular, Vena Cava, Superior / physiopathology, Cardiac Resynchronization Therapy Devices, Cardiovascular Surgical Procedures

## Introduction

Current guidelines clearly define the subset of heart failure patients who benefit from device implantation.^1^ Although first-time trans-venous device implantation has a high success rate, some patients present complex and challenging technical problems.^2^

## Case Report

**Figure 1:**
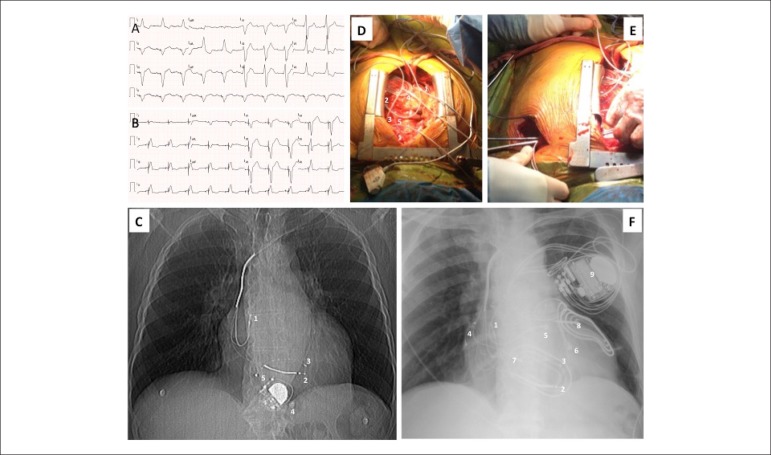
A) 12-lead ECG before epicardial CRT-D implantation: sinus P waves with dissociated right ventricular epicardial pacing; B) 12-lead ECG after epicardial CRT-D implantation: sequential atrial pacing and biventricular pacing. C) Chest x ray before epicardial CRT-D implantation: 1 Abandoned endocavitary right atrial lead; 2- Abandoned endocavitary right ventricular pacing/defibrillator lead; 3- Abandoned endocavitary left ventricular lead; 4 - Epicardial mono-chamber pacemaker generator; 5 - Epicardial mono-chamber pacemaker lead. D) Intra operatory situs after lead implantation: 1 - Epicardial right atrial lead; 2 - Epicardial right ventricular outflow tract lead; 3- Left ventricular lateral lead; 4- Epicardial anterior defibrillator patch; 5- Epicardial posterior defibrillator patch. E) Intra operatory situs showing lead tunneling to left sided pre pectoral pocket. F) Chest x ray after epicardial CRT-D implantation: 1 Abandoned endocavitary right atrial lead; 2 - Abandoned endocavitary right ventricular pacing/defibrillator lead; 3 - Abandoned endocavitary left ventricular lead; 4 - Epicardial right atrial lead; 5 - Epicardial right ventricular outflow tract lead; 6- Left ventricular lateral lead; 7 - Epicardial anterior defibrillator patch; 8 - Epicardial posterior defibrillator patch; 9 - Epicardial CRT-D generator. CRT-D: cardiac resynchronization and defibrillation; ECG: eletrocardiogram.

We present a case of a 73-year-old male patient admitted to our cardiology department for acute heart failure and two episodes of monomorphic ventricular tachycardia with hemodynamic collapse. Eight years previously the patient was diagnosed with class NYHA II heart failure, non-ischemic dilated cardiomyopathy with 32% left ventricle (LV) ejection fraction and complete left bundle branch block. After optimized medical therapy, he underwent conventional CRT-D implantation through the left subclavian (SC) vein in another institution. Two years later defibrillator lead fracture was diagnosed. The lead was abandoned and another defibrillator lead was implanted through the right SC vein and further tunneled subcutaneously to reach the left-sided prepectoral pocket. The procedure was complicated with superior vena cava thrombosis and device infection and the patient underwent right defibrillator lead and generator extraction. The previously implanted right atrial lead, fractured defibrillator lead and LV pacing lead were abandoned. One year later the patient was diagnosed with complete heart block and was submitted to epicardial mono-chamber pacemaker (VVI-R) implantation with supra-peritoneal epigastrium pouch (Figure 1C).

On admission at our department, 12 lead electrocardiogram (Figure 1A) showed sinus P waves with dissociated right ventricular epicardial pacing (Vp). Device interrogation revealed 99% Vp. Echocardiographic evaluation showed a dilated LV with severely depressed ejection fraction (20%) due to diffuse hipokinesia. Coronary angiogram confirmed the absence of coronary disease. Venous angio computed tomography demonstrated complete obstruction of the superior vena cava drainage system and severe fibrosis around the abandoned leads.

A surgical off-pump complete epicardial CRT-D implantation was decided. A median sternotomy was performed and complete epicardial CRT-D implantation was accomplished with a Starfish^®^ 2 heart positioner aid. The previously implanted epicardial mono-chamber pacemaker was extracted. A sutured bipolar lead [Capsure® Epi 4968 (Medtronic Inc., Minneapolis, Minnesota, USA)] was placed in the lateral wall of the right atrium (RA) and two sutureless screw-in bipolar leads (MyoDex® 1084T [St. Jude Medical Inc., Little Canada, Minnesota, USA]) were placed in the right ventricular outflow tract (RVOT) and lateral LV wall. Two defibrillator epicardial sutured patches were implanted in the anterior and posterior surface of the heart (Figure 1D). All these leads were then tunnelled to a left sided pre pectoral pocket and connected to the generator [Brava® CRT-D (Medtronic Inc., Minneapolis, Minnesota, USA)] - Figure 1E. Acute pacing parameters were excellent (RA - 1 mv/0,4 ms; RVOT - 2,5 mV/0,5 ms; LV - 2,5 mV/1,5 ms). Defibrillation testing was performed was performed at the time of implantation. Induced ventricular fibrillation was appropriately detected with successful defibrillation at 25J (Figure 1B, 1F). The patient remained for 24 hours in the intensive care unit and was thereafter transferred to the cardiology ward where he remained for 7 days before discharge under optimized medical therapy.

## Discussion

There were three alternative percutaneous approaches to complete CRT-D implantation in the presented patient: 1) lead extraction and left sided implantation; 2) implantation through the inferior vena cava system;^3^ 3) sub-xiphoid epicardial implantation.^4^^,^^5^ The Heart Team considered lead extraction unfeasible due to complete obstruction of the superior venous system and severe fibrosis around the abandoned leads. Implantation through the ileo-femoral vein and inferior vena cava system was considered high risk as it was the only venous draining site to the heart. Also, defibrillating vectors would be inadequate and the risk of lead dislodgement and infection high. Percutaneous sub-xiphoid epicardial access was considered unfeasible due to the presence of the previous epicardial pacemaker. In addition, supra-peritoneal generator placement would also produce inadequate defibrillating vectors.

Epicardial pacing and defibrillator systems have long existed and defibrillator coils are though to offer better long-term outcomes than defibrillator patches due to the high rate of patch crinkling (36-54%).^6^ This is associated with lead malfunction and chronic chest pain. However, access to epicardial defibrillator material is particularly difficult and in some countries only defibrillator patches are approved for epicardial usage.

Complete epicardial CRT-D implantation has been described in patients undergoing on-pump cardiac surgery for other reasons.^7^ Minimally invasive surgery using a small thoracotomy or using video-assisted thoracoscopy with or without robotic assistance is well described for LV lead implantation when a percutaneous procedure fails.^8^ A complete CRT-D has also been implanted using robotic assistance.^9^ Since there is no surgical access to the RV and RA, the RV lead was placed on the anterior wall of the LV and the RA lead in the left atrial appendage. Also, it is not possible to implant a defibrillator patch using this technique, and its availability is scarce. Although there is no cost-effective data regarding minimally invasive LV lead surgical implantation, it is well known that robotic assisted mitral valve repair is associated with greater costs.^10^

## Conclusion

To our knowledge, this is the first report of a complete off-pump epicardial sequential atrial-biventricular resynchronization and patch defibrillation device implantation requiring a median sternotomy. To clarify the effectiveness and safety of this procedure, more cases and longer-term observation are mandatory.
